# Effect of Workplace- versus Home-Based Physical Exercise on Muscle Response to Sudden Trunk Perturbation among Healthcare Workers: A Cluster Randomized Controlled Trial

**DOI:** 10.1155/2015/902896

**Published:** 2015-10-25

**Authors:** Markus D. Jakobsen, Emil Sundstrup, Mikkel Brandt, Kenneth Jay, Per Aagaard, Lars L. Andersen

**Affiliations:** ^1^National Research Centre for the Working Environment, Lersø Parkallé 105, 2100 Copenhagen, Denmark; ^2^Department of Sports Science and Clinical Biomechanics, SDU Muscle Research Cluster (SMRC), University of Southern Denmark, Campusvej 55, 5230 Odense, Denmark; ^3^Physical Activity and Human Performance Group, SMI, Department of Health Science and Technology, Aalborg University, Fredrik Bajers Vej 7, 9220 Aalborg, Denmark; ^4^Electronics and Computer Science, Faculty of Physical and Applied Sciences, University of Southampton, University Road, Southampton SO17 1BJ, UK

## Abstract

*Objectives*. The present study investigates the effect of workplace- versus home-based physical exercise on muscle reflex response to sudden trunk perturbation among healthcare workers. *Methods*. Two hundred female healthcare workers (age: 42 [SD 11], BMI: 24 [SD 4], and pain intensity: 3.1 [SD 2.2] on a scale of 0–10) from 18 departments at three hospitals were randomized at the cluster level to 10 weeks of (1) workplace physical exercise (WORK) performed in groups during working hours for 5 × 10 minutes per week and up to 5 group-based coaching sessions on motivation for regular physical exercise, or (2) home-based physical exercise (HOME) performed during leisure time for 5 × 10 minutes per week. Mechanical and neuromuscular (EMG) response to randomly assigned unloading and loading trunk perturbations and questions of fear avoidance were assessed at baseline and 10-week follow-up. *Results*. No *group by time* interaction for the mechanical trunk response and EMG latency time was seen following the ten weeks (*P* = 0.17–0.75). However, both groups demonstrated within-group changes (*P* < 0.05) in stopping time during the loading and unloading perturbation and in stopping distance during the loading perturbation. Furthermore, EMG preactivation of the erector spinae and fear avoidance were reduced more following WORK than HOME (95% CI −2.7–−0.7 (*P* < 0.05) and −0.14 (−0.30 to 0.02) (*P* = 0.09)), respectively. WORK and HOME performed 2.2 (SD: 1.1) and 1.0 (SD: 1.2) training sessions per week, respectively. *Conclusions*. Although training adherence was higher following WORK compared to HOME this additional training volume did not lead to significant between-group differences in the responses to sudden trunk perturbations. However, WORK led to reduced fear avoidance and reduced muscle preactivity prior to the perturbation onset, compared with HOME. This trial is registered with Clinicaltrials.gov (NCT01921764).

## 1. Introduction

Low back pain (LBP) is one of the most prevalent and costly work related health problems which affects millions of workers and workplaces worldwide [1–4]. Healthcare work is associated with an elevated risk of back pain and musculoskeletal injuries among women [5, 6]. Particularly the frequent often nonanticipated and high loadings of the spine while twisting and bending the back during patient handling [7–10] increase the risk for experiencing acute injuries and/or developing LBP among healthcare workers [11, 12].

Previous literature suggests that LBP alters muscle recruitment patterns. For example, numerous studies have shown that LBP is associated with delayed muscle reflex responses to sudden trunk loadings compared with healthy controls [13–15]. Accordingly, people with current LBP respond differently to sudden trunk loading than people without a history of LBP. In addition, reports of elevated preactivation levels as an attempt to stabilize the trunk prior to perturbation—maybe as a result of fear avoidance of sudden movement—have been observed in subjects with LBP [16]. However, no previous studies have investigated whether reductions in LBP are accompanied by improved (faster) muscle response to sudden trunk loadings. Nevertheless, a faster muscle reflex response implies an earlier stabilization of the spine [17] which may protect against overload injury. In support of this, Cholewicki et al. demonstrated, in a prospective study, that healthy subjects with a delayed lower back reflex response during sudden trunk perturbation had an increased risk of future low back injury [18]. Thus, improving trunk reflex response through, that is, exercise intervention may protect against future injury to the spine and truncus region.

Only a few studies have investigated the effect of training on the neuromechanical response to sudden trunk loading. In a 9-week longitudinal study Pedersen et al. trained healthcare workers without a previous history of LBP to react to a variety of sudden trunk loadings [19]. The training resulted in reduced trunk displacement and stopping time during unexpected trunk perturbations but did not alter reflex latencies measured with surface electromyography (EMG) in the erector spinae muscles. However, the reduction in trunk stopping time was accompanied by an increase in the neuromuscular (EMG) activity just prior to the instant where the perturbation was effectively stopped. Similar observations of increased EMG amplitudes have been demonstrated in the erector spinae muscle after 10 weeks of stabilizing exercise programs in patients with subacute recurrent LBP without any changes in reflex latencies [20]. Pedersen and coworkers showed, in a recent study, that 16 weeks of recreational soccer training significantly reduced trunk stopping time and stopping distance in healthy women compared with subjects who underwent continuous running exercise [21]. These authors concluded that the high number of sudden loadings (tackles, accelerations, and decelerations) exerted on the trunk during soccer training was responsible for the observed changes in stopping time and distance [21]. Accordingly, it was suggested that trunk exercise programs should not only focus on training trunk muscle strength and flexibility but also incorporate exercises with unexpected sudden loadings. In these studies the average training exposure was 2 times 45–60 min per week performed either before or after working hours which may be difficult and expensive in terms of working hours spent to incorporate as a part of the daily routine of a healthcare worker. Therefore, it remains to be investigated whether short-term physical exercise performed without a specific focus on unexpected trunk reactions, either at the workplace during working hours or at home, can improve trunk muscle response to sudden unexpected perturbations.

The present study investigates the effect of workplace- versus home-based physical exercise on muscle response to sudden trunk perturbation among healthcare workers.

## 2. Methods and Analysis

### 2.1. Study Design

This two-armed parallel-group, single-blinded, cluster randomized controlled trial with allocation concealment recruited female healthcare workers from three hospitals (18 departments) situated in Copenhagen, Denmark, was conducted from August 2013 to January 2014. To increase adherence and avoid contamination between interventions we chose to cluster-randomize the participants at the department level. The participants were allocated to a 10-week intervention period and randomly assigned to receive either workplace or home-based physical exercise. To ensure that the study aim, hypothesis, and primary outcome parameters were predefined the study was approved by The Danish National Ethics Committee on Biomedical Research (Ethical committee of Frederiksberg and Copenhagen; H-3-2010-062) and registered in ClinicalTrials.gov (NCT01921764) prior to enrolment of participants. The present study followed the CONSORT checklist to ensure transparent and standardized reporting of the trial. All experimental conditions conformed to The Declaration of Helsinki. Details on the study protocol and primary outcome variables (change in average muscle pain intensity of the low back, neck, and shoulder) have been published elsewhere [22, 23].

### 2.2. Recruitment and Randomization

The recruitment of participants was two-phased and consisted of a short screening questionnaire conducted in June 2013, followed by a baseline clinical examination and questionnaire performed in Aug-Sept 2013.

**Figure 1:**
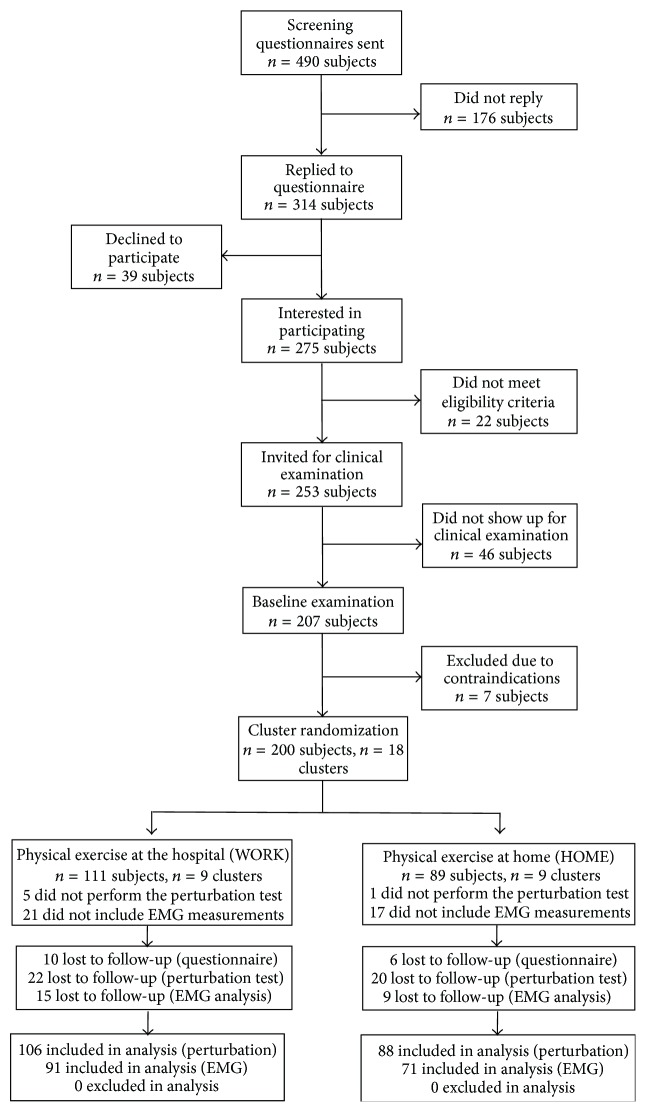
Participant recruitment flow-chart.

Initially, a screening questionnaire was administered to 490 healthcare workers (aged 18–67 years) from three Danish hospitals situated in Copenhagen in June 2013. Subsequently, in August and September 2013, a total of 207 female healthcare workers participated in the baseline clinical examination. Exclusion criteria were pregnancy and cardiovascular and life-threatening disease. The overall flow of participant enrolment, test, and EMG measurement is depicted in Figure 1 and has been described in detail elsewhere [22].

On the basis of the questionnaire we randomly allocated the 18 departments (200 participants), using a computer-generated random numbers table, to receive either physical exercise at the workplace or at home. The participants at each department and their management were informed by e-mail about group allocation. All examiners were blinded to the group allocation at 10-week follow-up and participants were carefully instructed not to reveal their particular intervention group. Baseline characteristics of the two intervention groups are listed in Table 1.

### 2.3. Interventions

Participants in each cluster were allocated to a 10-week intervention period receiving either physical exercise at the hospital or physical exercise at home. Both groups were encouraged to perform physical exercises for 5 × 10 minutes a week. The specific intervention protocols are briefly summarized below, since they are described in detail elsewhere [23].

### 2.4. Workplace Physical Exercise (WORK)

Subjects randomized to physical exercise at their workplace (WORK) (*n* = 111 subjects, *n* = 9 clusters) performed group-based and supervised high-intensity strength training using kettlebells, Swiss balls, and elastic bands (Thera-Band) exercises during working hours at the hospital (the exercises have been described in detail elsewhere [23]). All training sessions took place in designated rooms located at or close to the respective departments and all sessions were supervised by an experienced training instructor. The training program consisted of 10 separate exercises: kettlebell deadlifts, kettlebell swings, squeeze, lateral raises, golf swings, and woodchoppers using elastic tubing, abdominal crunches, back extensions, and squats using a Swiss ball, and lunges using elastic tubing. For each training session the instructor chose 4–6 exercises that were performed as circuit training, that is, with quick transitions from one exercise to the next using no or minimal periods of rest. Training intensity (loads) progression was ensured by using progressively more resistant elastic bands and heavier kettlebells throughout the 10-week intervention period, as supervised by the instructors. WORK furthermore offered 5 group-based motivational coaching sessions (30–45 min with 5–12 participants in each session) during working hours.

### 2.5. Home-Based Physical Exercise (HOME)

Participants randomized to home-based physical exercise (HOME) (*n* = 89 subjects, *n* = 9 clusters) performed physical exercises during leisure time at home. After the participants were informed about group allocation they received a bag with (1) training equipment (easy, medium, and hard elastic tubing) and (2) 3 posters that visually demonstrated the exercises that should be performed for the shoulder, abdominal, and back muscles and also contained recommendations for training progression [24–26].

### 2.6. Outcome Measures

#### 2.6.1. Assessment of Sudden Perturbation

**Figure 2:**
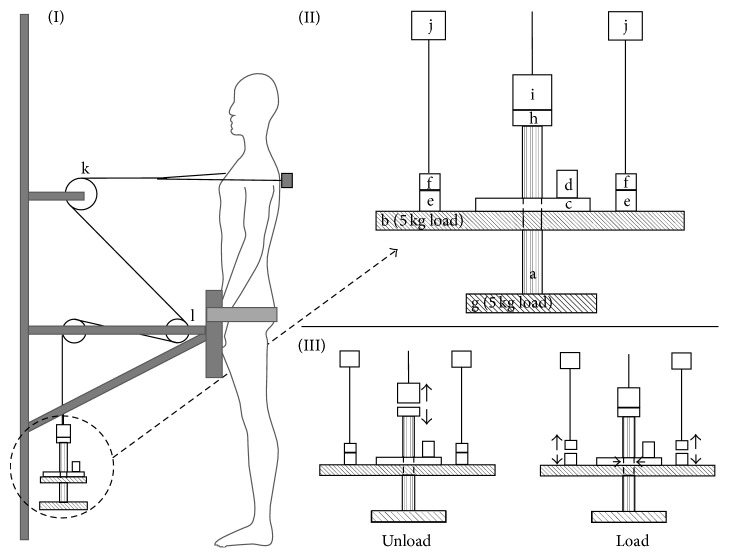
(I) Set-up for generating sudden perturbations to the upper part of the subject's trunk. The wire is fastened to a rigid bar fastened by a vest at the upper part of the trunk. The movement of the trunk is measured by a potentiometer mounted on a reel. (II) Details of the perturbation apparatus (right top: 90 degrees rotation) and standing position (left): (a) cylinder, (b) 5 kg load, (c) gripping device, (d) solenoid for activating gripping device, (e) holding magnets, (f) load-bearing construction, (g) 5 kg load, (h) anchor plate for magnet, (i) holding magnet, (j) bearing construction, (k) vertical adjustable reel with potentiometer, and (l) horizontal adjustable reel to adjust wire length to individual subject height. Generation of sudden unloading ((III) left): first the computer activates the magnet (i) and releases the load (b, h, and g) causing the weight of the load applied to the wire to suddenly decrease from 5.4 kg (a) to 0.1 kg (i). Generation of the sudden loading ((III) right): first the computer activates the solenoid (d) causing the gripping device (c) to fix the load (b) to the cylinder (a) and secondly deactivates the holding magnets (e). This releases the load (b, h, and g) causing the weight of the load applied to the wire to suddenly increase from 5.4 kg (a) to 10.9 kg (a–d).

Measurements of the neuromechanical reaction to sudden unexpected trunk perturbations were performed by the same examiner before and after the intervention period. The method used for the perturbation has been described in detail previously [27]. In brief, perturbations were generated by means of a special loading/unloading device wired to a rigid bar attached to the upper part of the subject's trunk at level of insertion of the deltoid muscle (Figure 2). The subject was standing with the front facing the initial load (5.5 kg) and the pelvis fixated against a wooden plate to allow only movement of the trunk. Subsequently, an increase (load: 10.9 kg) or decrease (unload: 0.1 kg) in the load was randomly applied to the subject. The perturbation events were triggered by a computer with a random delay between 5 and 25 seconds unknown to the subject and the investigator. The test protocol consisted of 6 randomized unloaded or loaded perturbations (3 of each). To avoid anticipation of the direction of the 6th perturbation the subjects were instructed that a range of 6–8 perturbations would be performed. A minimum of 45 seconds of rest was ensured between each trial. The linear movement of the subject's trunk was recorded using a potentiometer attached to a reel that steered the wire. Prior to the perturbation event the subject was instructed to stand as relaxed as possible in the initial position and subsequently informed that within 25 seconds they would experience a moderate perturbation and instructed to immediately resist the perturbation and return to the initial position.

**Figure 3:**
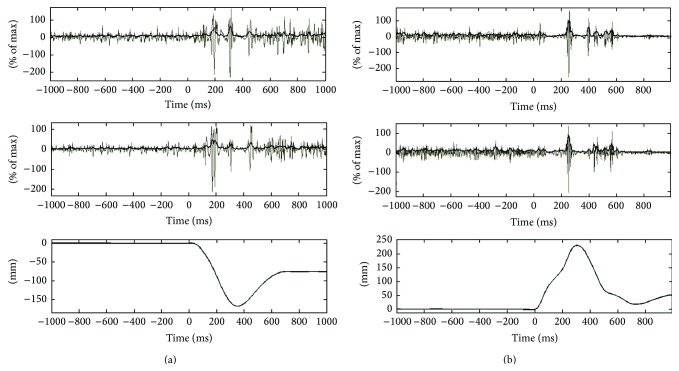
Potentionmeter and normalized back extensor EMG recordings of a (a) sudden trunk loading perturbation and (b) sudden unloading trunk perturbation. The perturbation was executed at time zero. The thin line is the raw signal and the thick line indicates the filtered EMG signal.

The analysis of the mechanical data obtained from the perturbations consisted of time elapsed from the onset of perturbation event until the movement of the trunk was reversed from perturbed direction (stopping time; time from event to maximum deviation of the movement curve from the initial position), distance moved from initial position to stop position (stopping distance) (Figure 3).

#### 2.6.2. Electromyography (EMG) Recording and Analysis

EMG activity was recorded (1024 Hz) bilaterally from the left and right erector spinae (longissimus). A bipolar surface EMG configuration (Blue Sensor N-00-S/25, Ambu A/S, Ballerup, Denmark) and an interelectrode distance of 2 cm were used [28]. Before affixing the electrodes, the skin of the respective area was prepared with scrubbing gel (Acqua gel, Meditec, Parma, Italy) to effectively lower the impedance to less than 10 kΩ. The electrodes were placed bilaterally at 2-finger width lateral from the processus spinosi of L1 (http://www.seniam.org/). The electrodes were fixated with tape (Fixomull stretch) and connected through thin shielded cables to a datalogger (Nexus10, Mind Media, Netherlands) that was placed in a flexible belt to ensure unrestricted mobility during the test.

The EMG signals were digitally high-pass filtered using a 10 Hz cutoff frequency (4th order zero-lag Butterworth filter). To remove electrocardiographic (ECG) artefacts we band-pass filtered (10–25 Hz) the raw signal and subtracted this signal from the high-pass filtered signal. The filtered signal was subsequently rectified and smoothed using a moving root mean square (RMS; 10 ms time constant). The filtered and smoothed EMG signals were normalized with respect to maximal muscle activity obtained during maximal voluntary contractions of the back extensors (described later). EMG signals assessed during the MVCs were filtered with a Butterworth 4th order high-pass filter (10 Hz cutoff frequency) and smoothed by a moving root mean square (500 ms time constant) [29]. Data filtering and data analysis were performed using custom-made Matlab programs (MathWorks).

The following EMG parameters were calculated to determine onset latency and the relative muscular load: The EMG onset latency [EMG latency] was defined as the time between the loading of the trunk and the EMG onset. EMG onset was determined as the point where the filtered signal for more than 15 ms exceeded preactivation with 1.4 standard deviations of the preload EMG activity (measured 1 s before loading) (this procedure was inspired by Radebold and coworkers [14]). Similarly, EMG shut-off latency [EMG shut-off] was defined as the point where the filtered signal was below 1.4 standard deviations of the preload activity (measured 1 second before loading) for more than 25 ms. Preactivation [EMG preactivation] was calculated as the mean EMG activity recorded within 1 second prior to the event (trunk loading/unloading). Postunload activation [EMG unload] was calculated as the mean EMG activity within the first 100 ms after the load was released. Finally, the maximal muscle activity obtained during the perturbation phase (from perturbation onset until perturbation was stopped) was identified as the peak-activation [EMG peak]. All EMG parameters where normalized to the maximal EMG activity measured during the maximal isometric back extension (MVC).

Synchronization of the EMG datalogger and the perturbation apparatus was ensured by a digital signal sent from the AD-converter (DAQCard-6036E, National Instruments) to the datalogger whenever a loading or unloading event occurred. A fixed 25 ms electromechanical time delay accounting for the triggering and loading/unloading was taken into account when calculating the EMG onset latencies. Figure 3 shows an example of the acquired data from a loading and unloading perturbation.

#### 2.6.3. Back Extensor MVC Testing

Maximal voluntary isometric contraction strength (MVC) was obtained for the lower back extensor muscles using a custom-built dynamometer with a strain gauge load cell (KIS-2, 2 KN, Vishay Transducers Systems). During the MVC maneuver the subject was standing in an upright position wearing a vest with a steel rod horizontally placed at the upper part of the back, at the level of insertion of the deltoid muscle [22]. At the distal end of the rod a wire was horizontally connected to a strain-gauge dynamometer. The subject was facing the dynamometer with the pelvis positioned against a wooden plate (upper edge aligned with the subject's iliac crest) while performing a maximal back extensor contraction (3 s) on a cue given by the tester. The participants performed 3 MVC attempts, separated by a 30-second rest period, while instructed to apply force to the dynamometer as fast and forcefully as possible. The maximum EMG signal (peak filtered EMG amplitude) of the 3 MVCs was used for subsequent normalization.

#### 2.6.4. Fear Avoidance

Participants were asked to reply to the following question at baseline and at 10-week follow-up immediately before performing a maximal and rapid isometric back extension: “Do you think that this rapid and forceful back extension will induce back pain or increase your back pain?” Subjects replied on a scale using 4 levels of fear avoidance (FA): “Not at all” (FA = 0), “A little” (FA = 1), “Some” (FA = 2), and “A lot” (FA = 3).

### 2.7. Statistical Analysis

All statistical analyses were performed using the SAS statistical software for Windows (SAS Institute, Cary, NC). The change in mechanical parameters (stopping time and stopping distance) and EMG (onset latency, shut-off, preactivation, unload, and peak) was evaluated using a linear mixed model (Proc Mixed) with* group*,* time*, and* group by time* as independent variables. Participant nested within department was entered as random effect. Analyses were adjusted for age and baseline values. All statistical analyses were performed in accordance with the intention-to-treat principle, that is, using the mixed procedure which inherently accounts for missing values. An alpha level of 0.05 was accepted as statistically significant. Outcomes are reported as between-group least mean square differences and 95% confidence intervals at 10-week follow-up. For all EMG parameters (onset latency, shut-off, preactivation, unload, and peak) mean values of the left and right erector spinae were selected for the statistical analysis [19]. Effect sizes were calculated as Cohen's *d* [30] based on the observed within-group changes (within-group changes from baseline to follow-up divided by the pooled standard deviation at baseline). According to Cohen, effect sizes of 0.20 are considered small, 0.50 moderate, and 0.80 large [30].

## 3. Results

### 3.1. Study Population

Baseline characteristics of the study participants are shown in Table 1. Participant flow is shown in Figure 1 and further described in detail elsewhere [22].

As described previously, training adherence differed between intervention groups (*P* < 0.001). Out of the 5 offered training sessions per week subjects in WORK performed on average 2.2 (SD: 1.1) sessions per week whereas subjects in HOME performed 1.0 (SD: 1.2) session [22].

### 3.2. Trunk Perturbation and Fear Avoidance

A priori hypothesis testing showed no* group by time* interaction for stop time and stop distance during the loading and unloading perturbation (*P* > 0.05) (Table 2). However, significant group by time interaction was seen for muscle pre-activation and unload-activation during the unloading perturbation (*P* < 0.05). There were no differences in peak-activation and EMG onset latency during the loading perturbation (*P* > 0.05).

We observed a group by time interaction for fear avoidance (*P* < 0.05); that is, HOME and WORK changed differently over time. At 10-week follow-up, a tendency (*P* = 0.09) for a difference in fear avoidance beliefs (−0.14 [−0.30 to 0.02]) was seen between WORK and HOME. Fear avoidance decreased (*P* < 0.001) from 0.71 [0.61 to 0.81] to 0.49 [0.38 to 0.59] following WORK whereas fear avoidance was unaltered (0.62 [0.52 to 0.73] to 0.62 [0.50 to 0.74]) following HOME.

## 4. Discussion

The present study demonstrated that ten weeks of low-frequency (2 sessions per week), short duration (10 min per session) physical exercise at the workplace is not superior to ten weeks of home-based exercise in reducing the muscle reflex response to sudden trunk perturbations among healthcare workers. However, compared to home-based exercise, greater reductions in muscle preactivation and fear avoidance were seen after physical exercise performed at the workplace.

Previous studies have demonstrated delayed muscle response to sudden trunk perturbations in patients with LBP compared with healthy controls [13–15]. Hence, the presence of pain may alter muscle recruitment. However, whether the impaired response to sudden perturbation is caused by the injury or pain itself or if the impaired neuromuscular function is a predisposing factor of pain remains unsolved. Damage to afferent receptors within the lumbar muscles and/or soft tissue of the spine could impair the magnitude and timing of somatosensory feedback from the trunk region to the CNS and in turn delay the reflex response. Pain may furthermore alter spinal neuronal excitability and thus negatively affect lumbar muscle activation [31, 32]. In addition, people with LBP may adopt an abnormal motor control strategy to avoid pain or to compensate for an injury or pain. Nevertheless, research is needed investigating whether reductions in musculoskeletal pain concurrently can reestablish concurrent impairments in motor control. In our study population, 10 weeks of workplace based exercise significantly reduced musculoskeletal back pain by 31% whereas smaller but nonsignificant changes (8%) were seen in response to 10 weeks of home-based exercise [22]. Despite these marked between-group differences in the effectiveness of pain reduction no between-group differences were observed for the mechanical (stopping time and distance) or neuromuscular (EMG onset latency) response to sudden trunk perturbation. Accordingly, it may be suggested that short-term reductions in perceived pain among subjects with mild to moderate pain intensity (average 3.1 SD 2.2 on a 0–10 scale) do not acutely alter motor control in response to sudden trunk perturbations, at least when achieved by means of low-frequency (twice per week) short-duration (10 min) exercise intervention.

Compared with ten weeks of home-based exercise, ten weeks of exercise at the workplace resulted in lowered preactivation of the erector spinae muscles immediately prior to trunk perturbation. There can be several explanations for this observation. Firstly, increased maximal muscle strength would result in a reduction in the relative magnitude of muscle loading for a given (fixed) perturbation load, hence potentially resulting in reduced levels of muscle preactivation. Accordingly, the 9% increase in maximal trunk extensor strength strength, shown in WORK [22], corresponds accurately to the 10% decrease in muscle preactivation observed in this group. Secondly, reports of increased muscle preactivation and elevated antagonist cocontraction levels as an attempt to stabilize the trunk and protect against injury and pain prior to perturbation have been observed in subjects with LBP [14, 16]. Thus, the reduction in pain in WORK may have decreased the subject's fear of injury and therefore contributed to the lower preactivation levels observed after the 10 weeks of workplace exercise. In support of this, a decrease in fear avoidance of rapid and forceful back movement was observed in WORK, suggesting that the subjects were less afraid of evoking lower pain or increasing their pain by performing fast and forceful muscle contractions. Whether this can also lead to more relaxed muscle activity patterns during the working day remains to be investigated. In female office workers with chronic neck and shoulder pain, when exposed to 10 weeks of strength training demonstrated a more relaxed muscle activity pattern throughout the working day [33].

Theoretically, lower levels of preactivation as seen following 10 weeks of workplace exercise may contribute to lower spinal stiffness thus potentially resulting in a larger perturbated trunk response. Hence, the lower levels of preactivation in WORK may have compromised the response to the sudden trunk perturbation. In addition, the HOME group demonstrated a tendency for an increase in preactivation which may have contributed to the faster stopping time and stopping distance during the loading and stopping time in the unloading perturbation observed in HOME. Consequently, these between-group differences in the change in preactivation should be taken into account when interpreting the present results on trunk perturbation.

Although neither of the present interventions were superior to the other in terms of stopping time and stopping distance several group by time differences emerged for the neuromuscular recruitment pattern during the perturbation task. During the unloading perturbation the WORK group demonstrated lower muscle activation in the first 100 ms after the instant of trunk unloading, compared with HOME. This suggests that the participants in WORK improved their ability to rapidly relax their trunk extensor muscles and/or to reduce the magnitude of cocontraction (the abdominal muscles are the primary muscles involved in stopping the trunk during the unloading) of the erector spinae muscles immediately after an unloading perturbation. Nevertheless, we did not see a group difference in how fast the muscle activity was reduced after the unloading as reflected by the nonsignificant change in EMG shut-off time. However, large negative within-group effect sizes for the EMG shut-off time indicate that a reduction was present in both groups, but large variations highlight the methodological challenges of evaluating this parameter using the present experimental setup. Nonetheless, delayed EMG shut-off time has been shown to increase the risk of future low back injury in healthy subjects [18]. Cholewicki et al. (2000), furthermore, proposed that measuring trunk stiffness and damping response to sudden loading of the trunk may provide an additional and more comprehensive understanding of the muscular patterns compared with the present calculations of mechanical displacement and timing [17]. Hodges and coworkers have moreover demonstrated that people with recurrent LBP have increased trunk stiffness and decreased damping [34]. It would therefore be interesting for future studies to investigate whether changes in low-back pain intensity, changes in recruitment pattern, and occurrence of low back injury are related with trunk stiffness and damping behavior.

Even though the present and previous studies report changes in EMG amplitude and/or stopping time and stopping distance in response to physical training [19–21, 35], training induced modifications in the EMG onset latency during sudden trunk loading remain to be demonstrated. Of notice, therefore, the participants in WORK demonstrated a tendency (*P* = 0.09) for a reduction in EMG onset latency which may have contributed to the faster stopping time that was observed during the sudden trunk loading perturbation following the ten weeks of workplace-based exercise.

We have previously shown, although using a slightly different test method, that 8 weeks of intensive kettlebell training (performed on average 2.1 × 20 min per week) significantly improved reaction to sudden unloading of the trunk, increased strength, and reduced musculoskeletal pain in laboratory technicians [36]. However, in the present study, only two out of the ten possible exercises in WORK involved the use of kettlebells. Consequently, the accumulated time the participants exercised with kettlebells might have been too little to induce similar improvements in trunk reaction. Moreover, besides increasing the volume of kettlebell training, incorporating exercises with unexpected trunk reactions, as suggested by Pedersen and coworkers [19], may have improved the perturbed response further.

Healthcare work involves high and unpredictable (nonanticipated) loadings of the spine [7–10] that may cause musculoskeletal pain and injury [5, 10, 37–39] in turn potentially leading to long-term sickness, improving the response ability to sudden trunk perturbations by means of exercise and physical training may be crucial for the working life of a healthcare worker. Accordingly, the within-group reductions in stopping time and stopping distance that were observed in WORK and HOME reflect a faster response capacity to counteract sudden unexpected trunk perturbations which may protect against future injury or pain.

As the within-group changes in trunk perturbation characteristics did not differ between groups a cost-effectiveness analysis would favor HOME as this represents a low cost intervention modality compared to the investment in working hours, instructors, coaches, and additional training equipment that is needed with WORK. However, if the aim of the intervention is not only to improve the response ability to trunk perturbation but also to reduce musculoskeletal pain and increase maximal muscle strength as seen in WORK [22], the workplace-based exercise intervention might be considered more favorable. Nevertheless, the overall summed effect of these different qualities needs to be evaluated in future cost-effectiveness studies.

### 4.1. Strength and Limitations

It may be considered a strength of present study that the test method, unlike those used in previous trunk perturbation studies, involved a random sequence of either loading or unloading perturbations. As the condition of the perturbation is unknown and therefore more difficult to foresee and thus to create a preprogrammed reflex pattern, this method may better resemble real life conditions such as unexpected trips or slips during patient handling.

A methodological limitation of the present study was that we only measured EMG on ~70% of all the participants (162 subjects). Nevertheless, the overall high number of participants in this study compared with previous studies investigating the effects of training on trunk stability with sudden perturbations definitely strengthens the validity of present observations. Yet, an even higher number of subjects would have increased the statistical power and may potentially have changed the tendencies to significant findings. Furthermore, the lack of an inactive control group may be viewed as a limitation of the study as it is difficult to say whether the within-group changes in both groups were caused by the physical exercise or alternatively caused by seasonal variations or reflecting a learning effect per se. However, disfavoring the possibility of learning effects, the detailed test-retest analysis of the present experimental methods did not reveal any significant learning effect within two weeks [22].

## 5. Conclusion

Although training adherence was higher when performed at the workplace (WORK) compared to exercising at home (HOME) this additional training volume did not appear to promote any between-group differences in the responses to sudden trunk perturbations. As the main findings of the study, however, significant within-group changes in both groups were seen for stopping time during both loading and unloading trunk perturbations and for stopping distance during unloading perturbations. Even though the relative perturbed load was reduced following for the intervention period as indicated by the lower preactivation levels of the erector spinae muscles in WORK, higher muscle strength does not necessarily result in faster reactions to sudden unknown trunk perturbations, at least when training is performed using low-frequency (2 session per week), short-duration (10 min) exercise sessions. Accordingly, exercise interventions aiming at improving neuromechanical trunk reaction ability should not only focus on increasing muscle strength but also contain elements that challenge coordination and trunk response.

## Figures and Tables

**Table 1:** Baseline characteristics of the two intervention groups. Values are means (SD).

	WORK	HOME
*N*	111	89
Age (years)	40^*∗*^ (12)	44 (10)
Height (cm)	168.4 (6.2)	168.0 (7.2)
Weight (kg)	67.5 (12.1)	68.9 (12.2)
BMI (kg·m^−2^)	23.8 (3.8)	24.4 (4.0)
Average pain intensity in the low back, neck, and shoulders during the last week (scale 0–10)	3.0 (2.2)	3.1 (2.3)

*∗* denotes difference between groups at baseline, *P* < 0.05. HOME: home-based physical exercise, WORK: work-based physical exercise.

**Table 2:** Baseline, follow-up, and between-group differences at follow-up and within-group effect size for the mechanical and the EMG parameters of the loading and unloading perturbation and preactivation EMG measured immediately before each perturbation. Values are means (95% confidence interval). All values are adjusted for baseline value.

		WORK						HOME						Differences at follow-up		
		0 weeks		10 weeks		*P*	Effect size	0 weeks		10 weeks		*P*	Effect size	Mean	95% CI	*P*
		Mean	95% CI	Mean	95% CI			Mean	95% CI	Mean	95% CI					
Load	Stopping time (ms)	362	(358–366)	352	(347–357)	<0.01	−0.23	360	(355–365)	351	(346–356)	<0.01	−0.21	1.2	(−6–8.4)	0.84
	Stopping distance (mm)	178	(174–181)	167	(164–171)	<0.01	−0.32	178	(175–182)	166	(162–170)	<0.01	−0.38	1.5	(−0.7–3.9)	0.52
	EMG onset latency (ms)	94	(90–98)	88	(83–93)	0.09	−0.12	95	(90–99)	95	(89–101)	0.90	0.01	−6.8	(−14.6–0.9)	0.22
	EMG peak (% of max)	81	(75–86)	83	(77–89)	0.51	0.07	81	(75–87)	89	(82–97)	0.08	0.22	−5.9	(−15.7–3.8)	0.37

Unload	Stopping time (ms)	371	(363–379)	358	(350–367)	0.02	−0.12	374	(365–383)	351	(342–361)	<0.01	−0.23	7.0	(−6.1–20.2)	0.18
	Stopping distance (mm)	263	(256–270)	258	(250–266)	0.27	−0.06	265	(257–273)	258	(250–267)	0.16	−0.09	−0.1	(−1.2–1.1)	0.75
	EMG shut-off (ms)	97	(77–116.5)	72	(47.9–96.5)	0.13	−1.42	93	(70.6–115.7)	79	(51–107)	0.44	−0.83	−6.6	(−43.8–30.6)	0.68
	EMG peak (% of max)	50	(48–53)	46	(43–50)	0.04	−0.14	51	(47–54)	47	(43–52)	0.17	−0.11	−1.1	(−6.4–4.3)	0.79
	EMG unload (%)	6.6	(6-7)	6.1	(5–7)	0.25	−0.11	6.6	(6-7)	7.5	(7-8)	0.08	0.20	−1.4	(−2.6–−0.2)	0.04

Pre	EMG preactivation (% of max)	9.1	(8.5–9.6)	8.1	(7.5–8.8)	0.02	−0.16	9.0	(8.4–9.7)	9.8	(9.1–10.6)	0.07	0.13	−1.7	(−2.7–−0.7)	<0.01

HOME: home-based physical exercise, WORK: work-based physical exercise. Negative effect sizes denote a within-group decrease from baseline to follow-up.
